# A Review of the Mechanism of Injury and Treatment Approaches for Illness Resulting from Exposure to Water-Damaged Buildings, Mold, and Mycotoxins

**DOI:** 10.1155/2013/767482

**Published:** 2013-04-18

**Authors:** Janette Hope

**Affiliations:** Environmental Medicine, 304 W. Los Olivos Street, Santa Barbara, CA 93105, USA

## Abstract

Physicians are increasingly being asked to diagnose and treat people made ill by exposure to water-damaged environments, mold, and mycotoxins. In addition to avoidance of further exposure to these environments and to items contaminated by these environments, a number of approaches have been used to help persons affected by exposure to restore their health. Illness results from a combination of factors present in water-damaged indoor environments including, mold spores and hyphal fragments, mycotoxins, bacteria, bacterial endotoxins, and cell wall components as well as other factors. Mechanisms of illness include inflammation, oxidative stress, toxicity, infection, allergy, and irritant effects of exposure. This paper reviews the scientific literature as it relates to commonly used treatments such as glutathione, antioxidants, antifungals, and sequestering agents such as Cholestyramine, charcoal, clay and chlorella, antioxidants, probiotics, and induced sweating.

## 1. Introduction

It has been estimated that up to 50% of illness results from exposure to indoor air pollution [1], with exposure to water-damaged indoor environments likely being a significant contributor to this. A number of treatment approaches have been used in the treatment of illness resulting from exposure to water-damaged buildings, molds, and mycotoxins [2, 3]. Symptoms and illness due to exposure result from varying mechanisms including infection, toxicity, allergy, irritant effects, and systemic inflammation. Additionally, individual responses to exposure vary based on genetic makeup, duration and severity of exposure, and underlying health and nutritional status. While it is often difficult to determine the contribution of the many components of water-damaged buildings, studies on illness from exposure to damp/water-damaged environments have been consistent in identifying the overall exposure itself as being the main factor associated with adverse health effects [4, 5]. Individual components of exposure that have been identified include: mold and mold spores, mycotoxins, bacteria, bacterial endotoxins and other cell wall components, protozoa (amoeba), increased presence of rodents, insects and dust mites, and increased deterioration of building materials with consequent offgassing of toxic fumes such as formaldehyde [4, 5]. While this paper focuses on individual mechanisms of illness for the purpose of reviewing treatment strategies, it is important to understand that the health consequences of exposure to water-damaged environments likely result from a combination of factors acting synergistically.

## 2. Molds and Mycotoxins

Amplified growth of mold in water-damaged, damp indoor environments contributes greatly to ill health effects and is extensively documented in the literature [4, 6–10]. Mold and mycotoxins are probably the best understood contaminants of water-damaged buildings and will be discussed throughout this paper. Exposure to water-damaged indoor environments has been shown to result in exposure to amplified growth of mold and mycotoxins including ochratoxin (OTA), aflatoxin, and trichothecene mycotoxins, all of which have been found in indoor environments [11–14] and in the bodies of those exposed to these environments [15–18]. Exposure to mold and mold components are well known to trigger inflammation, oxidative stress, and inflammatory reactions in both human and animal studies and have frequently been found in association with air found in water-damaged indoor environments [13, 19–26]. Thousands of mycotoxins have been identified to date; however, we will limit discussion to those currently felt to have the most relevance to health effects resulting from water-damaged indoor environments. 

Aflatoxins are produced by *Aspergillus parasiticus, Aspergillus flavus, Aspergillus nomius, *and various species of *Penicillium, Rhizopus, Mucor, and Streptomyces *[27]. Aflatoxin B1 (AFB1) is genotoxic, immunotoxic, hepatotoxic, mutagenic, and considered one of the most abundant, most toxic, and most potent naturally occurring carcinogenic substances known and is the leading cause of liver cancer in many developing countries [27–29]. Sterigmatocystin produced by multiple species of *Aspergillus* is considered only slightly less toxic than aflatoxin [29].

Ochratoxin A is produced by *Aspergillus ochraceus, Aspergillus niger, Aspergillus, *and species of *Penicillium, Petromyces, *and *Neopetromyces.* OTA is a nephrotoxic, genotoxic, immunotoxic, and [30] neurotoxic [22, 31] mycotoxin which is a known carcinogen in animals and a class 2B, possible human carcinogen. Associations have been found with human kidney disease [32, 33] including Balkan endemic nephropathy [34] and focal segmental glomerulosclerosis (FSGS) [35].

Trichothecenes are produced by *Stachybotrys, Fusarium, *and* Myrothecium* mold and include T2 toxin, deoxynivalenol (DON) and the macrocyclic trichothecenes satratoxin, and verrucarin [36]. Trichothecenes are distinguished by the presence of trichothecene ring and epoxide group at C-12 [37]. Trichothecenes are considered extremely toxic and have been used as biological warfare agents [37]. Much of the toxicity from trichothecenes is felt to result from inhibition of protein synthesis [36]. Trichothecene mycotoxins cause multiorgan effects including emesis and diarrhea, weight loss, nervous disorders, cardiovascular alterations, immunodepression, hemostatic derangements, skin toxicity, decreased reproductive capacity, and bone marrow damage [37]. A study of the trichothecene mycotoxin, satratoxin A, demonstrated that neurological system damage can occur in water-damaged buildings contaminated with fungal growth at levels that can occur indoors [38].

## 3. Bacteria and Endotoxins

Research continues to support the presence of bacteria and their components in water-damaged buildings. Gram positive bacteria found in water-damaged buildings often include species of *Actinobacter* including *Streptomyces*, *Mycobacteria,* and *Nocardia* which are capable of causing pulmonary diseases and other illnesses [39]. Gram negative bacteria, such as *Pseudomonas*, have also been identified in water-damaged buildings and are capable of causing illness through infections and the effects of endotoxins [39]. The extent to which an individual will be affected by exposure to bacteria and endotoxins will depend on the nature and severity of exposure as well as genetic makeup [40] and immune system function. Unfortunately, the immune system can be negatively affected by the exposure itself  [41, 42] which will worsen illness.

Endotoxins, often referred to by their active component, lipopolysaccharides are formed in the cell walls of gram-negative bacteria and can trigger a profound inflammatory response mediated by cytokine release. In a recent study of healthy human volunteers administered *E. coli* endotoxin intravenously, elevations in cytokines, particularly TNF*α*, were noted compared to baseline and the authors noted that several of the criteria for systemic inflammatory response syndrome were observed after endotoxin administration, including elevations core temperature, heart rate, and white blood cell (WBC) count, and noted elevations of epinephrine and cortisol [43]. Furthermore, studies have shown that endotoxins act synergistically with mycotoxins to enhance the cytokine-mediated inflammatory response [24, 44].

 Endotoxins have been found in water-damaged buildings with many of the studies occurring in flood-damaged homes in New Orleans. A study of homes affected by hurricanes Katrina and Rita showed that, in addition to elevated mold spore counts found in water-damaged homes, endotoxins and fungal glucans were detected at levels that can cause adverse health effects [45]. An additional study of eight flood-affected homes in New Orleans found endotoxins and beta-glucans and noted that smaller size particles (<1.0 and 1.8 microns) were found to have concentrations of endotoxin and glucans at the same level as particles greater than 1.8 microns [46]. One additional New Orleans study also quantified endotoxins indoors, however, at similar levels to the nonflooded homes also located in New Orleans [47].

Inhalational exposure to endotoxins has been found to be associated with physiologic abnormalities in animal studies. A study of intratracheal administration of lipopolysaccharide (LPS) to rat pups increased brainstem expression of inflammatory cytokine interleukin-1B (IL-1B) and is associated with impaired hypoxic ventilator response [48]. This could be particularly important in infants and young children as brain inflammation can not only have significant effects on early development but may play an important role in the development of neurodegenerative disorders later in life. Perinatal exposures to LPS have been shown to increase risk of dopaminergic disorders in animal models Parkinson's disease, autism, cerebral palsy, and schizophrenia [49]. A synergistic role of endotoxins and trichothecene mycotoxins is supported by studies in mice [24] as well as in human macrophages [44]. Endotoxins can also interact synergistically with other toxins worsening the consequences of exposure. In one study, LPS was injected intracerebrally into a neonatal rat to trigger brain inflammation and it was found that the application of the pesticide rotenone resulted in motor and neurobehavioral impairments in the rats exposed to LPS and not in the unexposed rats [50]. The authors speculated that perinatal brain inflammation may enhance adult susceptibility to environmental toxins. Similar results were seen in another study of exposure of neonatal rats to LPS. The authors concluded that the chronic inflammation resulting from exposure may represent silent neurotoxicity and that compromised dendritic mitochondrial function might contribute to this [49].

## 4. Microbial Volatile Organic Compounds (MVOCs) and Other Sources of VOCs

MVOCs are found in water-damaged homes. However, their role as a marker for mold contamination and as a significant cause of human illness is still being elucidated. Mold VOCs have remained difficult to measure. Even when deliberate mold contamination occurred in a climate chamber, only 9 VOCs were detected and the authors concluded that even under those favorable conditions the MVOCs are hardly accessible and were not a reliable indicator of mold growth in water-damaged indoor environments [51]. One study of 23 homes showed a higher level of certain MVOCs in basements compared to the main floor of the house although overall MVOC levels did not significantly differ [52]. A Japanese study evaluated MVOC exposure and clinical symptoms [53]. It found that the presence of MVOCs and airborne fungi was only weakly correlated and that higher levels of the MVOC 1-octen-3-ol increased irritation of the nasal and ocular mucosa. Offgassing of VOCs, such as formaldehyde, from water-damaged building materials can also pose a risk to health [4].

## 5. Routes of Exposure

While foodborne exposure to mycotoxins and fungal contaminants has been well researched, substantial information about airborne and transdermal routes of exposure also exists. Airborne exposure is likely the most significant route of exposure in water-damaged indoor environments; however, transdermal and potentially foodborne exposure through contact with indoor mycotoxins can also occur in these settings.

The airborne presence of mycotoxins has been well documented in research studies and has been reported to cause human illness throughout the medical literature. Respirable particles (<1.0 micron) represent the majority of particulate material found in indoor air [54] and mycotoxins have been found to be present on these indoor particles which include hyphal fragments [55]. Trichothecene mycotoxins have been found to be present in the air of buildings contaminated by *Stachybotrys* mold. In one study, macrocyclic trichothecene mycotoxins were measurable in the air and concentrations increased with sampling time and short periods of air disturbance [11]. These conditions imitate the nature of human exposure which is typically long term, with normal activity repeatedly causing air disturbance which increases aerosolization of mold and mycotoxins. Toxicity from inhaled mycotoxins appears to be very significant. In a study involving rats and guinea pigs, toxicity from inhaled T2 mycotoxin was 20 times as toxic as intraperitoneally administered toxin in rats and at least twice as toxic in guinea pigs [56]. Of significance in that study the pathologic lesions resulting from exposure were similar whether the exposure was inhalational or systemic. Experiments studying effects of acute inhalation of T2 mycotoxins in both young and mature mice showed that inhalation of T2 mycotoxins is at least 10 times more toxic than systemic administrations and at least 20 times more toxic than dermal administration [57]. Clinical symptoms seen in these animals immediately after exposure were tremors, lethargy, stilted gait, and in some cases prostration [57]. These are common symptoms seen in humans exposed to water-damaged buildings, mold, and mycotoxins.

Other studies support clinical and serological effects of inhaled mycotoxins. A study of 44 individuals exposed to indoor *Stachybotrys chartarum* identified the presence of trichothecene mycotoxins by ELISA in the sera of 23 individuals while only 1 of the 26 controls tested positive [18]. In goats exposed to macrocyclic trichothecenes mycotoxins by tracheal installation, mycotoxins were detected in the sera 24 hours after exposure at similar levels whether the goats were exposed repeatedly or to a single dose [58]. Ochratoxin, aflatoxin, and zearalenone have been detected in the air of a poultry house [59]. The authors quantified the amounts a worker in this setting may inhale and expressed concern about the potential public health consequences of this exposure, as it can affect workers directly exposed to mycotoxins and the quality of the food. One study of a problematic household where occupants were experiencing symptoms known to be associated with ochratoxicosis in farm animals, such as increased thirst, polyuria, edema, skin rash, and lethargy, found elevations of OTA on all surfaces tested at concentrations up to 1500 ppb which was found on a heating duct dust [14]. OTA has been found in dust from other indoor settings as well [60]. Of great clinical significance is the identification of mycotoxins on items found in human living environments including building materials, air filters, and personal items [13]. ELISA techniques have detected the presence of mycotoxins in persons exposed to water-damaged environments in a number of tissues including urine, nasal polyps and secretions, cancerous breast tissue, spinal fluid, breast milk, gastric and colon tissue, bladder and transitional cell carcinomas, brain astrocytoma, lung, lymph nodes, especially those with granulomatous diseases and renal cell tumors [16]. Mycotoxins have been found in breast milk by clinicians treating patients exposed to mold and mycotoxins in indoor settings. A study of 113 breast-feeding moms in Sierra Leone found the presence of OTA and aflatoxins at levels which in some cases far exceeded those permissible in animal feed in developing countries [61].

Intact spores are not the only source of aerosolized exposure. It has been shown that fungal fragments, often submicron-sized, can be released at 320 times higher level than spores and that the number of released fragments cannot be predicted based on the number of spores [62]. Increased reactivity of smaller fragments has been documented as they have the potential to penetrate deeper into the respiratory tract than intact spores which are generally greater than 2.5 microns [38, 62]. 

Biotransformation of mycotoxins in nasal mucosa can also play a significant role in the consequence of aerosolized exposure to mycotoxins. Nasal biotransformation of xenobiotics has been addressed in the literature as many biotransformation enzymes including cytochrome P450 1B1 and glutathione S transferase P1 have been detected in nasal mucosa of humans in levels at least as abundant as in the liver [63]. Rats exposed to intranasal aflatoxin B1 demonstrated high local bioactivation in the tissue and translocation of aflatoxin B1 and/or its metabolites to the olfactory bulb and also demonstrated mucosal injury [64].

Skin penetration of mycotoxins also occurs. Dermal contact with mycotoxin-contaminated items can also be a source of exposure which has the potential to occur even after a person has removed themselves from the contaminated environment since many people bring mold and mycotoxin-contaminated items to their new settings. One study showed that aflatoxin B1, OTA, citrinin, T2 toxin, zearalenone all penetrated human skin *in vitro* and that ochratoxin had the highest permeability [65].

## 6. Mechanisms of Illness

Illness resulting from exposure to water-damaged building can be caused by infection, toxicity, allergy, and inflammatory responses triggered by exposure to one or more of the agents present in water-damaged buildings and are often mediated by oxidative stress. Types of disorders that can be seen resulting from water-damaged environments, mold, mycotoxins and bacteria include, infections and mycoses, chronic and fungal rhinosinusitis, IgE-mediated sensitivity and asthma, other hypersensitivity reactions, pulmonary inflammatory disease, immune suppression and modulation, autoimmune disorders, mitochondrial toxicity, carcinogenicity, renal toxicity, neurotoxicity, and DNA adducts to nuclear and mitochondrial DNA causing mutations [39]. A significant mechanism of injury includes oxidative stress [23, 31, 66–69]. This becomes significant as it directs the approach to treatment, which focuses on removing ongoing sources of oxidative stress in the body, such as mycotoxins, as well as instituting treatments which focus on countering oxidative stress like glutathione and other antioxidants [70–74]. Inflammation triggered by exposure also appears to play a significant role in illness during and after exposure to water-damaged environments [24, 26, 75].

Most commonly, however, many mechanisms are interacting in an individual at any given time, making it imperative to address the illness with a comprehensive, multifaceted approach. Although respiratory symptoms are common from exposure to water-damaged indoor environments, it is important to note that a typical patient presents with multiple symptoms which are often debilitating, including fatigue, neurocognitive symptoms, myalgia, arthralgia, headache, insomnia, dizziness, anxiety, depression, irritability, gastrointestinal problems, tremors, balance disturbance, palpitations, vasculitis, angioedema, and autonomic nervous system dysfunction [76, 77]. The development of new onset chemical sensitivity is also commonly seen after exposure and can have a severe impact on a person's life [77].

It is always important to identify and address abnormalities that are found at increased frequency in persons exposed to water-damaged building such as thyroid, immune dysfunction and autoimmune conditions [78]. However, clinicians treating these conditions often see significant improvement with comprehensive treatment and detoxification [2].

Clearly, genetic individuality plays a role in response to exposure and response to treatment in ways that are still being elucidated. Human leukocyte antigen (HLA) patterns have been studied for their effects on response to exposure and genetic polymorphisms affecting detoxification pathways and inflammatory responses are also likely significant. For example, a human study looking at the effects of genetic polymorphisms on the effects of alpha tocopherol in 160 male volunteers evaluated polymorphisms in 15 pathways involved in inflammation or response to oxidative stress after exposure of peripheral blood mononuclear cells to lipopolysaccharides [40]. The authors found that the ability of alpha tocopherol to influence production of cytokines TNF *α*, IL *β*-6, and -10 was influenced by polymorphisms in GSTP 1 313 and genes encoding for TNF*α* and IL10 [40]. The study of genetic influences on illness and treatment after exposure to water-damaged buildings remains an exciting area for further research.

## 7. Neurocognitive Symptoms

Some of the most distressing symptoms encountered by patients following exposure to water-damaged indoor environments and toxigenic molds include neurocognitive disturbances. A disturbing study, conducted in Poland, measured IQ scores in children exposed to indoor mold for greater than two years, showed statistically significant IQ deficits in children exposed to indoor mold [79]. This study controlled for multiple variables and involved testing of 277 term babies at age 6 years using the WISC-R scale of intelligence and tests of neuropsychological function. Children exposed to indoor molds showed a statistically significant deficit of approximately 10 points. Additionally, it was shown in this study that longer exposure to indoor molds tripled the risk for low IQ scores defined as values below the 25th percentile. This is consistent with several other studies showing lower scores on cognitive testing [80, 80–83]. This is not surprising as several mycotoxins are known to be neurotoxic in animal studies including OTA, T2 toxin, macrocyclic trichothecene, and fumonisin [84]. Research has shown that satratoxin H can cause neurological system cell damage at levels found in water-damaged buildings, and it is believed that the constant activation of inflammatory and apoptotic pathways in human brain capillary endothelial cells, astrocytes, and neural progenitor cells can amplify the devastation of neurological tissues and lead to neurological system cell damage from indirect events triggered by the presence of trichothecenes [38]. Depression has also been shown to be increased in persons exposed to damp indoor environments [85]. Air quality can be a trigger for neuroinflammation as was seen in a highly disturbing study of healthy children and dogs exposed to outdoor air pollution. In this study the children exposed to the polluted air of Mexico City were compared to controls and found to have significant deficits on cognitive testing and 56% of the Mexico City children were found to have prefrontal white matter hyperintense lesions similar to those seen in the exposed dogs who were found to have significant neuroinflammation based on brain studies assessing levels of proinflammatory cytokines [86]. 

Patients who have developed symptoms as a result of exposure to mold and mycotoxins can present similarly with several classic neurologic disorders including pain syndromes, movement disorders, delirium, dementia, and disorders of balance and coordination [87]. Abnormalities have been seen on standardized neurocognitive test batteries [77, 81]. These disturbances frequently include disturbances of balance as determined by patient history, examination (Romberg with eyes open and closed, tandem gait, and balance standing on toes with eyes open and closed), and, ideally, with objective testing including computerized sway balance testing [81, 88]. Worsening of these symptoms on testing repeated months to years after initial exposure is frequently seen [81]. However, it remains unclear whether patients have truly removed themselves from further exposure by avoiding contact with items that had been present in the water-damaged home. Studies have also shown abnormalities in quantitative EEG (QEEG) studies [83] and single-photon emission computed tomography (SPECT) scans [77, 89] in patients exposed to mold and mycotoxins in indoor settings. Clinicians working with patients with neurocognitive symptoms resulting from exposure to water-damaged environments have seen improvement with the comprehensive treatment approaches outlined above including use of intranasal glutathione [2].

A review of mycotoxins found to be neurotoxic in animal models, macrocyclic trichothecenes, T2 toxin, fumonisin B1 (FB1), and OTA found that FB1 induces neuronal degeneration in the cerebral cortex, T2 induces neuronal cell apoptosis in fetal and adult brains, OTA causes depletion of striatal dopamine and its metabolites, and macrocyclic trichothecenes cause neuronal cell apoptosis and inflammation in the olfactory epithelium and bulb [84].

## 8. Oxidative Stress

Oxidative stress is being increasingly understood as a significant mechanism of illness from exposure to water-damaged buildings. For example, ochratoxin A, which is commonly found at elevated levels in persons exposed to water-damaged homes, is a known cause of cellular changes associated with oxidative stress. A recent study of human blood mononuclear cells exposed to OTA showed increased levels of reactive oxygen species and 8-hydroxydeoxyguanasine (8-OHdG), a marker of oxidative stress [21]. Additionally, in this study, OTA-induced DNA strand breaks, G1 phase arrest, and apoptosis of human mononuclear cells, contributing to the immunotoxicity of OTA. Of significance in this study, application of N-acetyl cysteine, a significant glutathione precursor, was able to significantly block some of the negative effects of OTA on the human cells studied.

Additional support for the role of oxidative stress comes from several studies of OTA. A study of rat kidney cells confirmed oxidative stress as a key source of OTA-induced DNA damage [67]. Support for oxidative stress-mediated impairments of mitochondria in rats has also been seen for aflatoxin B1 [28, 68]. Neurotoxicity from oxidative stress mechanisms has also been seen from macrocyclic trichothecenes, OTA, fumonisin B1, and T2 toxin [84].

## 9. Allergy, Autoimmune Responses, and Nonallergic Respiratory Disease

Respiratory illnesses have consistently been found to be associated with exposure to water-damaged, damp indoor environments [4, 6, 90]. Examples of this include chronic rhinosinusitis, allergic rhinitis including allergic fungal rhinitis, and sinusitis, asthma (new onset and exacerbations), conjunctivitis, invasive, and allergic pulmonary aspergillosis (ABPA), hypersensitivity pneumonitis, and sarcoidosis [9, 90–94]. It has been estimated that 21% of asthma in the United States is attributable to dampness and mold exposure [7], exposure to mold odors at home increased the risk of developing asthma in children 2.4 times [8], and exposure to workplace mold increased the risk of new-onset asthma 4.6 times [95]. It is important to note that studies have found that allergic response to mold is often not IgE mediated. A study of adult asthma found that those who developed occupational asthma were significantly more likely to have been exposed to water-damage and mold at work [96]. Interestingly, though, in this study, only 33.1% of the probable occupational asthma patients were atopic to any environmental antigen and only 20% were sensitized to mold allergens suggesting mechanisms other than type 1 allergy are involved in this symptomatology. Similar results were seen in a Mayo clinic study where a high prevalence of nasal fungal growth was found in both symptomatic rhinosinusitis patients and controls [97] with the most significant difference being the development of eosinophilic mucin and not type 1 hypersensitivity since IgE positivity was not seen in the majority of patients [97]. 

After sensitization, avoiding exposure to allergenic triggers and decreasing the immune response to unavoidable exposures are the main principles of treatment. Allergy to mold clearly places an individual in a damp and moldy indoor environment at increased risk for illness. Allergy testing to mold varies by individual examiner and ranges from comprehensive to minimal panels. Allergy to dust mite can also place an individual in a damp indoor environment at increased risk as dust mites have been shown to grow at amplified levels in damp environments [4]. While avoidance of exposure is always essential, allergy treatment techniques have been used with good success including injection and sublingual immunotherapy [3]. While not all illness resulting from exposure to water-damaged indoor environments is due to mold, some disease clearly is.

## 10. Infection and Colonization

Fungal infections can occur throughout the body and can be a result of exposure to water-damaged indoor environments. These have traditionally been felt to be associated with immunosuppression but have also been seen in immunocompetent hosts [98, 99]. Direct exposure to elevated levels of mold spores indoors can contribute to fungal disease, either directly by allowing seeding for fungal growth such as that occurring in nasal mucosa or by direct toxic effects and immune system alteration resulting from exposure. Additionally, some treatments received by patients due to illness resulting from their exposure, such as antibiotics and steroids, can contribute to fungal growth throughout the body including in nasal, sinus, and gastrointestinal tissue.

Nasal infections and colonization deserve discussion as many patients develop respiratory symptoms during and after exposure to water-damaged indoor environments. Untreated nasal and sinus infections can be a cause of ongoing symptoms and should be addressed. Chronic respiratory symptoms resulting from exposure can lead to the use of antibiotics and steroids which can predispose a person to fungal infections. Allergic fungal rhinitis has long been identified as a cause of persistent nasal infection, receiving initial attention with a Mayo clinic study which found fungal growth in 96% of patients with chronic sinusitis [97]. Types of molds identified on culture in this study included *Aspergillus, Penicillium, Alternaria, Cladosporium, Fusarium, Cryptococcus, *and many other types of mold known to produce mycotoxins or have the potential to cause significant disease.

Treatment approaches include avoidance of exposure to elevated spore counts and particulates through remediation and the use of air filters, sinus irrigation, topical antifungals, and antimicrobials which can include agents such as EDTA for aiding in breaking down biofilm and rarely oral antibiotics or oral antifungals. Surgery is indicated in refractory cases and in the presence of mycetomas [54].

The results of clinical studies of the use of topical antifungals have been mixed. A study of 51 patients with chronic rhinosinusitis who were treated with intranasal amphotericin B found improvement in nasal obstruction and discharge in 75% of the patients and 25% reported complete resolution of symptoms after treatment with improvement starting after one to three months. The only reported side effect was burning on application in 20% of the patients although no patients discontinued therapy due to side effects [100].

A placebo-controlled double-blind trial of intranasal amphotericin B showed symptoms improved with treatment; however, it did not differ from symptom improvement seen with saline irrigation alone [101]. Another randomized placebo-controlled, double-blind trial found that intranasal amphotericin B reduced nasal inflammatory mucosal thickening on computed tomography (CT) and endoscopy and reduced intranasal markers for eosinophilic inflammation in patients with chronic rhinosinusitis [102]. A Cochrane review concluded there was no benefit to topical or systemic antifungals for chronic rhinosinusitis [103] which is consistent with a more recent meta-analysis [104]. Intranasal ketoconazole, fluconazole, and itraconazole are frequently used. However, more data is available for intranasal use of amphotericin B. Oral antifungals have been used for allergic fungal sinusitis [105]; however it is advised to use caution with oral antifungals given the risk of significant side effects, especially in those with cytochrome p450 polymorphisms affecting the metabolism of these drugs. A study of children undergoing bone marrow transplant found the use of intranasal amphotericin B to be a safe and effective intervention for preventing invasive aspergillosis in pediatric patients undergoing bone marrow transplantation [106].

While genetic polymorphisms of glutathione S transferase enzymes have been found to play a role in a number of illnesses, including asthma, a study evaluating the role of these enzymes in chronic rhinosinusitis did not find a correlation [107].

Gut microflora play an essential role in the immune system and detoxification of xenobiotics [108, 109]. Alterations in the gut flora are increasingly being seen to contribute to illness including allergic and autoimmune diseases such as asthma, eczema, and rheumatoid arthritis [108, 109]. Exposure to water-damaged environments, mold and mycotoxins can result in injury to the gastrointestinal tract [22, 37] and, again, treatments often used to treat illness, such as antibiotics and steroids, can result in profound alteration in gastrointestinal flora, thus decreasing metabolism of mycotoxins and other toxins. Identifying and treating these abnormalities can be significant in restoring health. The use of both bacterial and yeast probiotics, treating infections, and identifying and avoiding food allergens can be significant steps in treatment [110–112].

## 11. Treatment Modalities

## 12. Avoidance and Total Load Reduction

The most important component of treatment is avoidance of further exposure to water-damaged environments and items contaminated by those environments as ongoing exposure will thwart any efforts at detoxification and perpetuate a reactive state. Unfortunately, it is often difficult and expensive to test environments and items that have been exposed to those environments for mycotoxin contamination [4] and consequently this testing is often not done. Research has shown that none of the commonly used methods for cleaning water-damaged materials such as bleach, ammonia, ultraviolet (UV) light, heating, and ozone were found to completely remove mold and mycotoxins from water-damaged building materials [113]. For this reason, it is safest for patients who have become ill after exposure to water-damaged environments is to avoid exposure to items that were present in the contaminated environment. Air spore counts are frequently done and, unfortunately, have significant limitations as they typically collect over a short (5-minute) period and can easily result in false negative results. The presence of elevation on spore count testing can have significance, however, both in terms of total spore count and types of mold present. The author of one study of schools concluded that a building must be considered unhealthy at spore counts over 1000 spores/m^3^ [114]. A study of a water-damaged hospital highlights limitations of traditional limited testing. The researchers measured multiple markers including culturable fungi and bacteria, endotoxin, submicron-size particles, and markers of fungi (extracellular polysaccharides specific for *Penicillium *and *Aspergillus*, ergosterol, and beta-1–3 glucans) and found the presence of submicron-sized particles and markers of microbiological agents was positively associated with a building with historic water-damage and higher prevalence of reported occupant symptoms [115]. The authors proposed that marker compounds in air and floor dust samples may be better indicators of health risk than culturable fungi or bacteria in air or settled dust. 

While abnormal test results can confirm the presence of a significant problem, they cannot be relied upon to ensure an environment is safe for rehabitation. Testing that can be useful in some situations includes environmental testing for bacteria and endotoxins, mycotoxins, VOCs and polymerase-chain-reaction (PCR) based mold testing such as ERMI to identify species of mold. It is important to note that an individual who had become ill in a water-damaged environment will likely be most sensitized to that specific environment and items present in the environment and may never be able to return to that environment or be exposed to items from that environment without getting ill. The ability of persons to return to a building without developing symptoms remains the most relevant indicator that a building has been properly remediated [116].

Unfortunately, common building remediation techniques have not been found to be successful in removing mold and mycotoxins from contaminated materials [113]. In this study, pine and gypsum were deliberately contaminated with *Stachybotrys chartarum* and *Aspergillus versicolor* and treated with either peroxide, hot air, flaming, two types of boron-based chemicals, drying, steam, UV light, ammonium chloride, or sodium-hypochlorite-based chemicals. No remediation treatment eliminated all the mycotoxins from the building materials. The study showed that none of the 10 different mold remediation agents and methods tested was able to totally remove mold from the infected materials and that they were ineffective in destroying mycotoxins. The authors conclude that there is a risk of inhaling mycotoxins in buildings even after mold remediation. Although not completely successful, boron, and ammonium-chloride-based chemicals were the most successful in reducing mold and mycotoxin levels. A common misperception is that killing mold, which is a relatively easy task, eliminates risk from contaminated environments or items. Unfortunately, this does little to decrease the risk as nonviable fungal spores, fragments, and mycotoxins remain present and, due to their structure, such as with an epoxide ring, [117] they can be extremely difficult to destroy. 

As previously noted, mycotoxins can travel not just on spores, but on fungal fragments which can often be submicron-size [39, 62], allowing inhalation deep into lung tissue and preventing complete protection from occurring with the use of a protective mask. Paper and soft materials are particularly vulnerable and cannot be adequately remediated even with washing and HEPA vacuuming and often need to be replaced [116].

It is also important to be aware that disturbing contaminated material can significantly increase exposure to spores and mycotoxin-contaminated fragments [11], dramatically worsening exposure during attempts of remediation or packing of items. The potential for dermal penetration of mycotoxins is also important to keep in mind [65] during any contact with contaminated materials.

In addition to avoidance of further exposure to contaminated items, it is recommended to decrease exposure to other chemical xenobiotic agents including pesticides, heavy metals, volatile organic compounds and fragrances, vinyl chloride, plastics, perflourinates (nonstick cookware), and other toxins in an effort to reduce total load and improve the ability to detoxify from the exposure to a water-damaged environment. It is common for patients exposed to water-damaged indoor environments to become sensitive to and avoid many chemicals which frequently becomes noticeable after leaving the environment.

A review of the CDC's fourth national report on human exposure to chemicals showed that acrylamides, cotinine, trihalomethanes, bisphenol A, phthalates, chlorinated pesticides, triclosan, organophosphate pesticides, pyrethroids, heavy metals, aromatic hydrocarbons, polybrominated diphenyl ethers, benzophenone from sunblock, perfluorocarbons from nonstick coatings, and several polychlorinated biphenyls and solvents were found in the majority of individuals tested [118]. All of these potential exposures as well as other toxic exposures are important to address when treating patients.

## 13. Glutathione

Given the role of oxidative stress in illness from exposure to mold and mycotoxins, the use of glutathione and glutathione precursors plays a large part in treatment. Glutathione is an endogenously produced tripeptide (glycine, cysteine, and glutamate) that in its reduced state functions in several enzyme systems in the body to assist in the detoxification of fat-soluble compounds and as an antioxidant, quenching free radicals [119]. Many disease states including Alzheimer's, Parkinson's disease, and autism have been found to be associated with low glutathione levels and have been treated with glutathione precursors (N-acetyl cysteine and whey protein) or various forms of glutathione [120–126]. One study found a correlation of low brain GSH levels with negative symptoms of schizophrenia [127].

Glutathione deficiency, as is frequently seen by clinicians treating patients exposed to water-damaged buildings, can have far-reaching effects on the body. In addition genomics testing often shows abnormalities in glutathione transferases including GSTP transferase and the GSTM null genotype which has been found to be associated with increased toxicity from aflatoxin [128]. Marked glutathione deficiency induces cellular damage associated with severe mitochondrial degeneration in a number of tissues [129] and glutathione deficiency results in mitochondrial damage in the brain [130]. Glutathione deficiency leads to widespread mitochondrial damage which is lethal in newborn rats and guinea pigs. Ascorbate and glutathione function together in protecting mitochondria from oxidative damage [131].

Reduced glutathione (GSH) can be administered in an intravenous, nebulized, transdermal, oral liposomal, and nasal form.

Nebulized glutathione is the only known treatment for increasing glutathione levels in the epithelial lining fluid, thought to be one of the first lines of defense for oxidative stress [119]. Beneficial effects have been seen for a number of conditions including cystic fibrosis [132–134], emphysema [135], chronic otitis media with effusion [136], idiopathic pulmonary fibrosis [137], chronic rhinitis [138], and HIV disease [139]. Reduced glutathione is known to be decreased in the bronchoalveolar lavage (BAL) fluid of persons with cystic fibrosis [140]. Inhaled glutathione was shown to decrease the proinflammatory prostaglandin E2 and increase CD4+ and CD8+ lymphocytes in BAL [140] which is consistent with an observational study which showed improvement in FEV1 and body weight and a decline in bacteria cultured including *Pseudomonas aeuruginosa* [134]. An additional cystic fibrosis study showed improvement in FEV1 and that GSH levels in BAL fluid improved by 3 to 4 times 1 hour after inhalation and remained doubled 12 hours after inhalation [133]. The author of one review of nebulized and aerosolized glutathione concluded that there were many potential applications for its use given the number of conditions related to deficient antioxidant status and impaired host defenses and with theoretic uses that included farmers lung, pre- and postexercise, cigarette smoking, and chemical sensitivity [119].

Caution should be used, however, as there is evidence of increased bronchospasm seen in some asthmatics as noted in one small study [141]. In this 8-person study, bronchoconstriction was felt to be provoked by sulfite formation and was blocked by nebulized salbutamol given before nebulized GSH. Since bronchoconstriction is felt to occur primarily in sulfite-sensitive asthmatics, it has been suggested by one author that testing of sulfites in urine occurs before nebulized glutathione therapy [119]. Additionally, obtaining a history of sensitivity to sulfites in wines or dried fruits can be useful. It is important to note that sulfite sensitivity is different from sensitivity to sulfonamide antibiotics, which is felt to be, at least in part, a result of decreased glutathione levels in HIV patients [142]. A practice that is commonly used in clinical practice requires the initial dose of nebulized glutathione to be administered in the office. In the absence of bronchoconstriction after this dose, home therapy is typically initiated. While this risk of increased bronchospasm clearly exists in select patients, there are no reported cases of status asthmaticus or death resulting from the use of nebulized glutathione.

Since olfactory epithelium is the only place where dendritic processes are directly exposed to the environment in the cribiform plate of the ethmoid sinus, intranasal delivery of medications can bypass the blood-brain barrier [143]. It has long been studied as a means to achieve central nervous system effects and has been studied for multiple agents in an effort to treat diseases such as depression, schizophrenia, Alzheimer's and Parkinson's disease [144]. Medications administered intranasally have reportedly been detected in the cerebrospinal fluid (CSF) as fast as 1 minute after delivery [144]. Intranasal administration of neuropeptides has been studied and found to have the advantage of bypassing the blood-brain barrier, which has served to limit the effectiveness of systemic therapies on central nervous system (CNS) symptoms. A study of 36 humans administered insulin, vasopressin, and melanocortin (MSH/ACTH) intranasally found that they received direct access to the CSF within 30 minutes, bypassing systemic circulation, as measured by intraspinal and intravenous catheters [145]. Levels of these neuropeptides were found in the CSF within 10 minutes and remained increased for up to 80 minutes. More prolonged sampling of a subgroup of patients receiving MSH/ACTH and vasopressin intranasally found that levels of these neuropeptides in the CSF remained above that of placebos (administered intranasal saline) at 100 to 120 minutes after administration and the authors believed that intranasal administration of neuropeptides could be useful for the treatment of brain diseases such as Alzheimer's disease and obesity.

Given the powerful antioxidant properties of reduced glutathione, clinicians have taken advantage of the transnasal delivery route and have used intranasal glutathione to successfully treat neurocognitive symptoms resulting from exposure to water-damaged buildings [2, 13]. Additionally, reduced glutathione is a powerful antioxidant which can have significant beneficial effects on the nasal mucosa. A study of the use of nasal glutathione found it increased GSH levels in the nasal mucosa and produced statistically significant improvement in nasal obstruction, rhinorrhea, and ear fullness [138]. In a recent survey study of 70 patients who had used intranasal glutathione for conditions that included multiple chemical sensitivity, allergies/sinusitis, Parkinson's disease, Lyme disease, fatigue, and other symptoms, over 86% of the respondents found intranasal glutathione nasal spray to be comfortable and easy to use and 62.1% reported experiencing health benefits while 12.1% of respondents reported having experienced adverse effects, the most common of which were irritation of nasal passages, headache and bloody nose [146]. A recent rat study of intranasal milnacipran, a serotonin noradrenaline reuptake inhibitor used for depression and fibromyalgia, found both CSF and systemic levels of the drug to be higher when administered intranasally compared to orally and demonstrated an increased antidepressant effect from the transnasal administration [144]. There is evidence for carrier-mediated transport of glutathione across the blood-brain barrier in rats administered GSH via carotid artery catheterization [147] and in other animal models [148]. Increasing CNS delivery of antioxidants including glutathione remains an area of much interest given the broad role oxidative stress is felt to play in neurologic and neurodegenerative diseases including the neurocognitive deficits frequently seen as a result of exposure to water-damaged buildings, mold, and mycotoxins [149].

Studies have shown that cerebral GSH plays an important role in maintaining blood-brain barrier function. A rat study showed that glutathione depletion was associated with blood-brain barrier dysfunction and that treatment with N-acetyl cysteine, methionine, and GSH provided a partial to full protection against GSH depletion and blood-brain barrier dysfunction, but treatment with *α*-tocopherol, ascorbic acid and turmeric was not effective [150]. Brain protection through the blood CSF interface involves a glutathione-dependent barrier system [151] and ischemic alterations of the glutathione redox system may unmask blood-brain barrier permeabilizing actions of leukotrienes [152] which could contribute to neurocognitive symptoms.

Glutathione at a low molecular weight of 307 Daltons appears to be a good candidate for intranasal delivery as studies have shown that the apparent cutoff weight for intranasal delivery is 1000 Daltons, with smaller molecules having better absorption [153]. The addition of the bioadhesive chitosan was seen to increase drug delivery into the CSF and plasma with increased drug effect, presumably due to increased residence time of the drug in the nasal cavity [144], a concept which is intriguing as a possibility of increasing the effect of intranasal medications including glutathione.

There is currently a phase 1 human study of nasal glutathione for the treatment of Parkinson's disease being conducted at Bastyr University [154].

Encapsulation in liposomes allows for systemic delivery of oral liposomal glutathione. An *in vitro* model of Parkinson's disease in rats showed that liposomal GSH was 100 times more potent than nonliposomal GSH at replenishing intracellular GSH [155]. Of interest in this study was that liposomal glutathione spared endogenous glutathione with exposure to paraquat plus maneb (used to induce Parkinson's disease) but did not require GSH biosynthesis for protection and no toxicity was seen at 200 times the half maximal effective concentration (EC (50)) needed for protection. A study in mice showed that oral liposomal glutathione had antioxidative and antiatherogenic properties towards macrophages [156]. Liposomal encapsulation was found to greatly increase bioavailability in a study involving administration of radioactive cobalt 60 to rats [157]. In this study orally administered liposomal glutathione was found to decrease Cobalt 60 levels in all tissues by 12–43% while the nonliposomal glutathione did not.

Transdermal glutathione is also an effective delivery method and may be particularly desirable in children, who may be less compliant with other methods. A clinical trial of the use of transdermal glutathione in children with autism demonstrated that the treatment group showed significant increases in plasma-reduced glutathione but not whole blood glutathione [158]. 

It is best to use GSH in conjunction with sequestering agents as administration of GSH appears to allow the mobilization of toxins, including mycotoxins as evidenced in a case of a woman with documented presence of mycotoxins who developed a reversible choreiform movement disorder after she discontinued sequestering agents and was using GSH for six weeks at high dose without sequestering agents [159]. Of note in this case was that levels of urinary mycotoxins increased dramatically during the time she was taking GSH without sequestering agents compared to when she was using both GSH and sequestering agents. 

## 14. Sequestering Agents

Sequestering agents refer to nonabsorbable materials capable of binding toxins in the gastrointestinal tract, thus reducing enterohepatic recirculation and ultimately the body burden of toxins. These agents are not absorbed into systemic circulation; therefore, side effects are typically limited to gastrointestinal symptoms and potential malabsorption of medications and nutrients, especially if the dose is poorly timed. Sequestering agents have a large surface area to volume ratio, giving large absorptive capacity. Several agents have shown specific efficacy in lowering mycotoxin and endotoxin levels including cholestyramine, activated carbons, and chlorella. Additionally, these agents are nonspecific and can bind additional toxins, helping to lower total body burden of toxins. Of course they have the potential to bind medications, vitamins, and nutrients and should be taken several hours apart from medications and vitamins and ideally on an empty stomach.

Mycotoxins are sequestered in a variety of tissues and enter enterohepatic circulation [132, 160, 161]. For example, OTA has been found in liver, muscle, fat, as well as the adrenal medulla and cortex, skin, gastric mucosa, myocarium, and bone marrow in animals [162]. 

A review of the literature shows a successful use of a variety of sequestering agents. Activated carbons (charcoal) have long been used medically, both for acute and delayed effects of toxins. Studies of use of activated carbons for mycotoxins have shown several beneficial effects. An *in vitro* study of activated carbons' binding capacity to OTA and deoxynivalenol found them to have a high affinity for chemically different mycotoxins [163]. The authors felt they could be considered multimycotoxin sequestering agents. Another study of 14 absorbent materials to detoxify *Fusarium* mycotoxin deoxynivalenol and nivalenol found only activated carbon to have effective binding capacity and to produce a significant reduction in intestinal mycotoxin absorption [164]. A recent study of nanodiamond substrates found them to be comparably effective for absorption of mycotoxins and comparable to activated charcoal while outperforming clay for OTA absorption [165]. In contrast, a study of aflatoxin binders in cows showed good results for sodium bentonite and an esterified galactomannan, while showing no effect from activated carbon [166]. An *in vitro* study of charcoal and cholestyramine for endotoxin removal showed they were both effective with both agents removing about 90% of the endotoxin from solution [167]. Cytokines have also been shown to be removed by sequestering agents with charcoal and silica found to be more effective at removing the cytokines ILF beta 1 and TNF alpha than cholestyramine [168].

Clay has been extensively studied for its effect on reducing toxicity from aflatoxin exposure, with the sodium montmorilonite clay Novasil being frequently studied. A randomized human trial of the use of Novasil studied over 600 blood and urine specimens from Ghana and found significant reductions in aflatoxin B1 adducts and a decrease in urine aflatoxin M1 at both doses used at 3 months [169]. Another review of the use of clay for the prevention of aflatoxicosis in animals found that Novasil clay binds aflatoxin with high affinity and capacity in the gastrointestinal tract resulting in noticeable reduction in aflatoxin bioavailability without interfering with the utilization of vitamins and other micronutrients including vitamins A and E, iron, and zinc [170, 171].

Chlorophyll and chlorophyllin, a water-soluble derivative of chlorophyll, have been found to be well studied as anticarcinogenic agents and have beneficial effects against aflatoxin toxicity. In a series of rat studies, Siminochich et al. showed that chlorophyl and chorophyllin provided potent chemoprotection against early and late biochemical and pathophysiological biomarkers of aflatoxin B1-induced carcinogenesis in rat livers and colons [172]. Chlorophyllin has the potential to reduce carcinogenicity of aflatoxins as it binds to aflatoxins and reduces their bioavailability which consequently significantly reduces AFB biomarkers in humans [173]. Caution should be exercised in the sourcing of marine-based supplements given the unfortunate known contamination of oceans with toxins and heavy metals. A recent study of contamination of natural supplements found contamination of some supplements including marine-sourced supplements like chlorella [174]. Techniques involving the growth of chlorella on filtered water would likely avoid this contamination and could provide a good option for treatment. Testing has shown at least one commercially available brand of chlorella to be free of contaminants [17].

Cholestyramine (CSM), an anion exchange resin that works as a bile acid sequestering agent, has been widely studied for its role in reducing a variety of toxins [175–177] including mycotoxins. CSM has generally been found to be safe and well tolerated even in children [178]. It has the ability to reduce enterohepatic recirculation of fat-soluble toxins and thus can be found throughout the literature as a treatment for many toxin exposures including mycotoxins and endotoxins. Animal studies involving the use of CSM for OTA exposure have shown it accomplishes the therapeutic goal of reducing plasma levels of OTA while enhancing fecal excretion of the toxin bound to CSM. A study was able to demonstrate decreased nephrotoxicity in rats exposed to OTA that were given CSM, by reducing plasma and urine values of OTA while increasing fecal excretion [179, 180]. An *in vitro* study showed the CSM had a higher affinity for OTA than bile salts and it was proposed that, in addition to effects on the enterohepatic circulation of OTA, alteration of the biliary bile salt pool may be a means in which CSM reduces OTA toxicity [181].

CSM has also been shown to bind endotoxins and was found to be more effective at removing endotoxin than charcoal or silica in an *in vitro* study [168] and has shown beneficial effects in preventing the suppression of the cellular immune system in rats after partial hepatectomy [182]. CSM has also been used in the treatment of infectious and noninfectious diarrhea including *Clostridium difficile* diarrhea [183, 184] and has been shown to be effective in the treatment of infectious diarrhea in the newborn with an *in vitro* study of CSM showing immediate binding of the toxins of *Vibrio cholera* and three strains of *E. coli* at a pH comparable to intestinal pH [185].

Clinicians typically treat with a combination of sequestering agents taken together 2 to 4 times a day, apart from medications and supplements [2].

## 15. Antioxidants and Nutritional Agents

In addition to glutathione, additional antioxidants and vitamins can be helpful. Patients seen in practice have often been ill for a prolonged period of time and identifying and correcting nutritional deficiencies essential for optimal detoxification and recovery. Common deficiencies encountered include vitamin D, magnesium, zinc, coenzyme Q10, and B vitamin deficiencies all, of which can adversely affect multiple pathways in the body necessary for detoxification, thereby perpetuating the effects of the toxin exposure. 

Multiple animal studies have tested the effect of nutritional supplementation to counteract effects of cellular damage caused by oxidative stress and mycotoxins. A study of rats exposed to aflatoxin B1 found that multiple cellular changes including deoxyribonucleic acid (DNA) fragmentation and lipid peroxidation which resulted from aflatoxin could be restored towards normal with the use of whey protein concentrate, Korean ginseng, or a combination of whey protein concentrate and Korean ginseng, although they did not fully reverse the effects of aflatoxins. [73]. It was suggested by the authors that genotoxicity from aflatoxin can be prevented by supplementation of whey protein, Korean ginseng or their combination. Whey protein supplies cysteine, the rate limiting step in glutathione synthesis. Animal experiments showed that the concentrates of whey proteins exhibit anticarcinogenesis and anticancer activity through their effect on increasing GSH concentration in relevant tissues and may have antitumor effects [186]. Whey protein is used routinely for treatment of illness resulting from exposure to mold and mycotoxins both as a glutathione precursor and as an easily absorbed protein source for patients, many of whom have developed significant gastrointestinal symptoms as a result of their exposure.

Melatonin and licorice extract (*Glycyrrhiza glabra*) were evaluated for their effect on OTA-induced oxidative stress and histopathological damage in the testes of male rats [187]. Serum total antioxidant power and total thiol molecules were assessed and found to be decreased in OTA-exposed rats, while those that received melatonin or Glycyrrhiza glabra extract showed an enhancement in these levels. In this study, significant histopathologic changes were also seen in the OTA-exposed rat testes including testicular degeneration, seminiferous tubular atrophy, and vasodilation with vascular thrombosis and both melatonin and Glycyrrhiza glabra was found to have protective effects against these changes. It was proposed by the authors that the antioxidant effects of these agents exerted a protective effect agains OTA-induced oxidative stress. An additional rat study evaluated the antioxidant effects of melatonin and coenzyme Q10 in rats exposed to a single high dose of OTA. Malondialdehyde and glutathione levels were measured and kidneys and bone marrow were examined. Malondialdehyde levels were significantly higher and glutathione levels were significantly lower in OTA-exposed rats compared to controls or those OTA-exposed rats receiving melatonin or coenzyme Q10. It was concluded by the authors that a single dose OTA administration caused oxidative damage and that melatonin or coenzyme Q10 appeared to ameliorate the OTA-induced tissue injuries [71]. Licorice was again evaluated for its effect on OTA-induced nephrotoxicity in rats [72]. In this study, rats exposed to 28 days of OTA showed elevated levels of serum creatinine, blood urea nitrogen, alkaline phosphatase, alanine aminotransferase, and malondialdehyde while the antioxidant power of the serum was significantly reduced and histopathological evaluation showed degenerative symptoms in proximal tubules, congestion in renal tissue, and remarkable infiltration of inflammatory cells. Liquorice plant extract was found to alleviate most of the biochemical alterations from OTA.

Melatonin was also evaluated for its role in aflatoxicosis in chicks [188]. The pathological changes seen in the aflatoxin-exposed chicks (vacuolar degenerations, necrosis, bile duct hyperplasia in liver, and mild tubular degeneration in kidney) were markedly reduced in the chicks given melatonin concurrently with their aflatoxin exposure. Additionally, it was noted that GSH levels were decreased and malondialdehyde levels were increased in aflatoxin exposed chicks, but with melatonin coadministration, the levels approached those of the controls. The authors concluded that the results indicated that nitrosative tissue degeneration induced by aflatoxin exposure could be greatly reduced by melatonin supplementation in chicks. An additional study of melatonin use in chicks exposed to aflatoxin B1-contaminated diets demonstrated that aflatoxin-exposed chicks showed an increase in lipid peroxidation in the liver, erythrocytes along with suppression of superoxide dismutase and catalase enzyme activities of erythrocytes, significant reduction of serum proteins, elevations in serum transaminases and decreasing of the humoral and cell-mediated immune responses in growing chicks. Simultaneous administration of melatonin showed an obvious improvement in all test parameters although long-term melatonin administration was more effective than short-term administration for countering aflatoxin B1-induced toxicity [74].

Vitamins A, C, and E were studied on human lymphocyte cultures exposed to a single dose of aflatoxin B1 with or without the addition of vitamins A, C, or E [70]. The experiment showed that aflatoxin B1 significantly reduced the level of GSH and activities of superoxide dismutase and glutathione peroxidase while increasing levels of malondialdehyde and that simultaneous supplementation with vitamins A, C, and E restored the parameters to normal range. It was felt that vitamins A, C, and E exhibited protective effects on human lymphocytes by inhibiting aflatoxin B1-induced reactive oxygen species generation. Additional support for the role of oxidative stress comes from a rat study involving the induction of glutathione deficiency by administration of L-buthionine-(S,R)-sulfoximine which was found to also decrease ascorbate levels in the kidney, liver, brain, and lung [189]. In this study, the administration of large doses of ascorbate to these glutathione-depleted rats decreased mortality, led to normal levels of ascorbate, and spared glutathione. 

Cultured human lymphocytes exposed to aflatoxin B1 were assessed for the presence of chromosomal aberrations and sister chromatid exchanges after treatment with varying doses of resveratrol. The number of sister chromatid exchanges and micronuclei was reduced in the presence of resveratrol resulting in decreased genotoxicity of aflatoxin B1 [190]. In contrast, a study in aflatoxin B1-exposed rats, showed that resveratrol failed to protect against aflatoxin B1-induced liver injury [191]. In the same study, however curcumin showed a significant hepatoprotective activity by lowering the levels of serum marker enzymes and lipid peroxidation and by elevating the levels of reduced glutathione, superoxide dismutase, catalase, and glutathione peroxidase. 

## 16. Probiotics and Dietary Interventions

Probiotics and various dietary interventions have been studied for their effects on modulating effects of toxins including mycotoxins. These treatments have the potential to have significant beneficial effects as much of the metabolism of toxins occurs via intestinal biotransformation. Ochratoxin A (OTA) undergoes hydroxylation to the less toxic ochratoxin alpha in the intestines. In fact, administration of radio labeled OTA to rats showed that effective metabolism of OTA was lacking in most tissues other than the intestines [192]. Antimicrobials can have a significant negative effect on gastrointestinal flora and detoxification process as was demonstrated in rats that were administered neomycin which resulted in decreased hydrolysis of OTA to ochratoxin alpha with elevated levels of OTA [193].

An *in vitro* study of various bacterial probiotics showed a reduction in bioaccessibility of Aflatoxin B1 and OTA with the use of these probiotics [194]. Fermented milk containing *Lactobacillus rhamnosus* GG and *Lactobacillus casei* strain Shirota alone and in combination with chlorophyllin demonstrated that the use of fermented milk with or without chlorophyllin was found to have a significant hepatoprotective effect against aflatoxin B1 by enhancing activities of glutathione-S transferase, glutathione peroxidase, catalase, and superoxide dismutase and lowering the levels of thiobarbituric acid-reactive substances [112].

The ability of *Lactobacillus plantarum* and *Lactobacillus rhamnosus* GAF01 to degrade or bind aflatoxin M1 *in vitro* was studied in mice [111]. They found both agents were able to remove aflatoxin M1 with superior removal being seen by *Lactobacillus rhamnosus*. Removal appeared to be by simple binding and the bacteria/aflatoxin M1 complex was stable and only a very small proportion of the mycotoxin was released back in solution. An additional study in quail exposed to aflatoxin B1 found the probiotic *Berevibacillus laterosporus* prevented the biochemical changes of decreased serum albumin, total protein, glucose, and cholesterol levels as well as the increase in serum uric acid, urea, creatinine, and phosphorus that was normally seen in the quail exposed to aflatoxin B1 [195].

A study of the yeast probiotic *Saccharomyces boulardii* in boiler chickens exposed to OTA showed improvement in the biochemical profiles of the *Saccharomyces boulardii*-treated group when compared to the untreated group which showed decreased values of total protein, albumin, and globulin and increased levels of serum creatinine and SGPT [110].

The *Fusarium* trichothecene mycotoxin, deoxynivalenol (DON) has been reported to be completely biotransformed by ruminal and intestinal microflora and eubacterium BSSH 797 was capable of DON degradation and counteracted the toxic effects of DON [196]. Additional studies on DON in crops have looked at promising effects from bacterial enzymes. 

Some data exists on the ability of diet-derived factors to influence aflatoxin B (AFB) biotransformation and some dietary factors efficiently protect from AFB-induced genotoxicity with mechanisms including the induction of detoxification enzymes such as glutathione-S transferases (GST) [173]. Consideration is given in this review to dietary components that may decrease the rate of activation of AFB by inhibiting cytochrome p450 1A2 activity, which has been shown to occur in humans with apiaceous vegetables (carrot and parsley family) as well as sulforaphanes, which are found in cruciferous vegetables and have been shown to protect animals from AFB-induced tumors, to reduce biomarkers of AFB in humans *in vivo,* and to reduce AFB adduct formation in human hepatocytes, most likely through repression of human hepatic 3A4 expression [197]. A randomized clinical trial involving the use of a broccoli sprout tea in China showed a significant decrease in aflatoxin adducts in individuals receiving the tea [198].

A study in rats exposed to aflatoxin B1 showed that pretreatment and intervention with lycopene significantly reduced the toxic effect caused by AFB(1) and greatly modulated AFB(1) metabolism and metabolic activation, decreasing the urinary excretion of AFB(1) phase 1 metabolites, AFM(1), AFQ(1), and AFP(1) serum AFB(1)-albumin adducts. This was felt to be a result of inhibition of phase 1 metabolism and metabolic activation as well as induction of phase 2 detoxification by lycopene [199].

Phloretin, a natural phenol found in apple leaves, has been shown to have beneficial effects against aflatoxin with a strong chemopreventive effect against AFB1 through its inhibitory effect on CYP1A2 and CYP3A4 and its inductive effect on GST activity [200].

The identification of food allergies and avoidance of problematic foods are also beneficial. Gluten deserves special mention as it can contribute to an inflammation [201] and neurologic and psychiatric symptoms [202–204]. Beneficial effects can be seen from the avoidance of gluten, even in those not found to have celiac disease [205].

## 17. Sauna, Exercise, Weight Reduction

Sauna and sweat induction have been used safely in many cultures throughout history and have long been studied as a means of reducing the body burden of toxins [206]. The most frequently studied saunas are Finnish dry heat radiant saunas, although far infrared saunas are also frequently used effectively and have the advantage of potentially inducing sweating at a lower body temperature. Sauna has been found to have numerous benefits including the treatment of respiratory and cardiovascular diseases [207]. Sauna therapy has shown benefit for the treatment of hypertension, congestive heart failure, and post-myocardial infarction (MI) care and has also been used effectively for chronic obstructive pulmonary disease, chronic pain, rheumatologic disease, chronic fatigue, and addictions [207].

Clinical studies have shown that sauna was safe for patients with stable heart conditions (hypertension, coronary disease, and stable controlled chronic heart failure) [208], and some studies have shown benefits for persons with cardiac disease including congestive heart failure and hypertension [206, 207]. Studies of the risk of sudden cardiac death have not found an increased risk of sudden cardiovascular death except when alcohol was used [207]. Contraindications to sauna therapy include pregnancy, concurrent use of alcohol, unstable angina, aortic stenosis, severe orthostatic hypotension, fever, oozing skin conditions, urticarial, or recent myocardial infarction, although there are some studies supporting the safe use of saunas in persons with a recent history of myocardial infarction [209]. Sauna use was not only found to be safe but actually transiently improved pulmonary function in a study of men with obstructive pulmonary disease [210].

A study of 28 persons exposed to mold and mycotoxins included treatment with exercise, physical therapy and sauna as well as well as IV antioxidants, oxygen therapy and immunotherapy found improvement in all patients with 27 of the 28 returning to work [3].

Dr. Stephen Genuis has studied the excretion of a number of agents in his blood, urine, and sweat (BUS) studies and has found that a number of toxins are found in sweat, with some appearing to be preferentially excreted in sweat [211]. He has identified bisphenol A (BPA) in sweat, even, in some instances when it was not identified in blood or urine, supporting the use as of sauna as a possible means to induce excretion of BPA. The presence of phthalates and their metabolites have also been identified in sweat [212] as have heavy metals [213]. Ochratoxin has been found in human sweat [17]. Controlled studies to evaluate for the presence of mycotoxins in sweat would be useful. However, regardless of whether mycotoxins are found, induced sweating will likely reduce the total overall body burden of toxins and support recovery in persons made ill from exposure to water-damaged buildings.

Exercise, whether or not sweating is induced, can have numerous physiologic benefits and should be encouraged at whatever level is tolerated. At least in rats, exercise is shown to prevent oxidative stress and memory deficits with chronic cerebral hypoperfusion [214] and also reduces oxidative stress in hyperphenylalaninemic rats [215]. There are numerous benefits to exercise, and it should be initiated at whatever level is tolerated and gradually increased. Deconditioning, often severe, is frequently seen in those suffering from chronic illness, including illness resulting from exposure to water-damaged buildings. A gradual, escalating approach to resuming exercise can be of great benefit in reversing this.

Weight gain is an unfortunate consequence of chronic symptoms resulting from long-term exposure to water-damaged indoor environments and can hinder health recovery. Obesity has been found to be associated with oxidative stress in humans and mice [216].

## 18. Conclusions

The treatment of patients who have become ill as a result of exposure to water-damaged buildings involves a comprehensive treatment approach utilizing available nutritional and detoxification strategies. Complete removal from exposure and contaminated items cannot be emphasized enough although it is often not sufficient for some people to regain health. Persistence of symptoms after exposure does occur, unfortunately, and is most likely related to genetic and nutritional factors as well as the severity, duration of exposure, and persistent exposure through cross contamination. The treatment approaches include the use of sequestering agents, antioxidant support, systemic, nebulized and intranasal glutathione, probiotics, nutritional support, and the correction of persistent fungal infections or symptomatic colonization. Also, the use of sauna and exercise can be invaluable in helping to restore the health of those injured from their exposure.

In 1989, the Massachusetts Department of Public Health estimated that indoor air pollution accounted for up to 50% of all illness [1]. It is likely this has increased since then and it would be expected that water-damaged indoor environments would be a significant contributor to this. Unfortunately, in spite of growing recognition of illness resulting from exposure to water-damaged environments, limited educational opportunities currently exist for medical students and residents to learn about the diagnoses and management of exposure-related conditions. Improving medical education as it relates to both indoor and outdoor air pollution would be a significant step in improving the quality of medical education and care for our patients. 

Clearly much more research is needed to identify the best treatment options for these patients; however, an attempt was made to present the research currently available that addresses treatment options available to physicians. Limitations to the current research include the fact that many of the studies are limited to animals or are relatively small human studies or case reports. While some large studies in humans have occurred with promising results, great benefit could come from further investigation into the safety and efficacy of treatment options available to physicians treating patients with the complex array of symptoms that often result from exposure to water-damaged buildings. Since many of the agents used in the treatment of persons exposed to water damaged indoor environments are readily available and non-patentable, funding for large scale studies is often unavailable. It is important to note that a typical human exposure involves a complex mixture of biocontaminants, while much of the research occurs on single agents. Similar limitations exist to studying treatment outcomes, as typically patients have been ill with multiple symptoms for a prolonged period of time and are understandably eager to proceed with treatment, pursuing multiple treatment options concurrently. As understanding of illness resulting from exposure to water-damaged building increases, it is hoped that research into the best treatment approaches will allow physicians to provide optimal care for their patients.
